# Automated Synthesis of ^**18**^F-Fluoropropoxytryptophan for Amino Acid Transporter System Imaging

**DOI:** 10.1155/2014/492545

**Published:** 2014-07-20

**Authors:** I-Hong Shih, Xu-Dong Duan, Fan-Lin Kong, Michael D. Williams, Kevin Yang, Yin-Han Zhang, David J. Yang

**Affiliations:** Department of Cancer Systems Imaging, The University of Texas M. D. Anderson Cancer Center, Houston, TX 77030, USA

## Abstract

*Objective*. This study was to develop a cGMP grade of [^18^F]fluoropropoxytryptophan (^18^F-FTP) to assess tryptophan transporters using an automated synthesizer. *Methods*. Tosylpropoxytryptophan (Ts-TP) was reacted with K^18^F/kryptofix complex. After column purification, solvent evaporation, and hydrolysis, the identity and purity of the product were validated by radio-TLC (1M-ammonium acetate : methanol = 4 : 1) and HPLC (C-18 column, methanol : water = 7 : 3) analyses. *In vitro* cellular uptake of ^18^F-FTP and ^18^F-FDG was performed in human prostate cancer cells. PET imaging studies were performed with ^18^F-FTP and ^18^F-FDG in prostate and small cell lung tumor-bearing mice (3.7 MBq/mouse, iv). *Results*. Radio-TLC and HPLC analyses of ^18^F-FTP showed that the Rf and Rt values were 0.9 and 9 min, respectively. Radiochemical purity was >99%. The radiochemical yield was 37.7% (EOS 90 min, decay corrected). Cellular uptake of ^18^F-FTP and ^18^F-FDG showed enhanced uptake as a function of incubation time. PET imaging studies showed that ^18^F-FTP had less tumor uptake than ^18^F-FDG in prostate cancer model. However, ^18^F-FTP had more uptake than ^18^F-FDG in small cell lung cancer model. *Conclusion*. ^18^F-FTP could be synthesized with high radiochemical yield. Assessment of upregulated transporters activity by ^18^F-FTP may provide potential applications in differential diagnosis and prediction of early treatment response.

## 1. Introduction

Molecular imaging agents play a major role in drug discovery and development because of their ability to quantify drug properties* in vivo*. For example, positron emission tomography (PET) agents show high specific activities since they are made through a nuclear transformation and use carrier free forms of isotopes. Thus, PET agents do not produce detectable pharmacologic effects but provide important information concerning the characterization of various diseases such as central nervous system diseases (epilepsy, psychosis, dementia, and Alzheimer's disease) [1–4], cardiovascular diseases (myocardial viability) [5], cancer staging, restaging, and treatment planning for the malignant diseases [6]. In addition, molecular imaging helps to control and monitor dosage for increased safety and effectiveness. The trends for PET agent development in oncology are to assist in the determination of optimal therapeutic dosing, delineation of differential diagnosis between inflammation/infection and recurrence, determination of sensitive or resistant to treatment response, and prediction of treatment response by selecting patient who may respond to therapy. ^18^F-Fluorodeoxyglucose (^18^F-FDG), a gold standard for PET, has been successfully used to image tumors with high glycolytic activity [7]. However, ^18^F-FDG has several limitations that give rise to false positive/negative diagnosis and poor predictive value of chemoradiation therapy to tumor response [8]. For instance, ^18^F-FDG has poor contrast in brain tumor due to the high uptake of glucose in normal brain tissue [9], and it has poor differentiation of tumor from inflammatory tissue because of high uptake of ^18^F-FDG in granulocytes and macrophages [10]. Radiolabeled amino acid is an alternative in characterizing tumors because of low accumulation in normal tissue, rather high accumulation in tumor tissue, and rapid blood clearance. Large neutral amino acids are uptaken by system L amino acid transporters (LAT), whose subtype LAT1 is reported to be upregulated in most cancerous tissues at both primary and metastatic sites [11–13]. For instance, 5-hydroxy-L-tryptophan (5-HTP) is taken up by the tumor cells via their LAT1 [14]. Upregulated LAT1 indirectly measures cell proliferation activity [15], and elevated expression of LAT1 in cancers correlates with poor prognosis [16].

Tryptophan is essential for normal growth and development in children as well as for keeping the nitrogen balance in adults. In addition, T-cells also depend on tryptophan for their immune response after invading cells have been recognized. Tryptophan is oxidized by indoleamine 2,3-dioxygenase (IDO) via the kynurenine pathway, and low uptake of tryptophan by LAT1 transporter may cause the T-cells death and subsequently result in cancers and autoimmune diseases. A high level of IDO expression reduces the concentration of tryptophan in the local tissues and starves T-cells for tryptophan. This results in an excess of kynurenine in the body fluids, which is toxic to T-cells and causes tumor immune resistance [17]. Tryptophan-kynurenine pathway plays a major role in neurological diseases and cancers [17, 18]. In addition, serotonin is also derived from tryptophan via tryptophan hydroxylase (TPH). Increased expression of TPH is associated with malignant phenotype in tumors. It has been suggested that IDO, LAT1, and serotonergic markers could serve as potential molecular targets to inhibit tumor cell growth [19]. Thus, it is amenable to develop a radiolabeled tryptophan to monitor* in vivo* expression and activity of IDO, LAT1, and TPH1 as well as to assess therapeutic response after selective inhibitors of IDO, LAT1, and TPH1 in cancer treatments.

Previously, *α*-[^11^C]methyl-L-tryptophan (AMT), a substrate for TPH and IDO, was developed to study tryptophan transporter system. In PET studies of brain tumors, elevated tumor AMT metabolic rates were associated with increased expression of IDO [19]. However, the short half-life of ^11^C (*t*_1/2_ = 20 min) makes it difficult in repeated imaging and multiple clinical studies. The half-life of ^18^F is 110 min which allows repeated imaging and is suitable for multiple clinic studies. Krämer et al. evaluated the* in vitro* and* in vivo* characteristics of 5-(2-^18^F-fluoroethoxy)-L-tryptophan (^18^F-FEHTP) as a PET probe for tumor imaging [20]. He et al. developed a similar analog 5-(3-^18^F-Fluoropropyloxy)-L-tryptophan (^18^F-FPTP) and performed its biologic evaluation [21]. ^18^F-FPTP was synthesized manually with a two-step reaction. The overall uncorrected radiochemical yield of ^18^F-FPTP was 21.1 ± 4.4% with a synthesis time of 60 min. Both ^18^F-FEHTP and ^18^F-FPTP were accumulated in cancer cells. ^18^F-FEHTP has been studied in endocrine (small cell lung cancer cells) and nonendocrine (PC-3, MDA 231) tumor models via LAT1 transporter system. ^18^F-FPTP was accumulated in hepatocellular carcinoma cell (Hepa 1-6) via the amino acid transport system B0+, LAT2, and ASC. ^18^F-FEHTP and ^18^F-FPTP were not decarboxylated by aromatic l-amino acid decarboxylase (AADC) and did not incorporate into protein synthesis [20, 21]. PET imaging revealed good uptake of ^18^F-FEHTP and ^18^F-FPTPin endocrine and nonendocrine tumors in rodents with low background. Though both ^18^F-FEHTP and ^18^F-FPTP could serve as PET probes for assessing tumor LAT1 activity, ^18^F-FPTP was superior to ^18^F-FEHTP and ^18^F-FDG due to its better differentiation between tumors and inflammation in animal models. ^18^F-FPTP had high tumor to inflammation count density ratio (2.53) at 60 min after administration [21].

Radiosynthesis of ^18^F-compounds must be rapid because of higher risk of radiation exposure and short half-life of ^18^F used during radiosynthesis. An automated apparatus is needed to assure production efficiency and minimize the radiation exposure by PET isotopes. ^18^F-FPTP was selected as a candidate for automation due to its superior quality of images compared to ^18^F-FEHTP. At present, commercially available black boxes are only suitable for production of ^18^F-FDG but not for the preparation of ^18^F-labeled tryptophan. To avoid mixing up or contamination, we developed a dedicated module for production of ^18^F-FPTP for clinical application. Here, we report the efficient automated synthesis of ^18^F-FTP (known as manually developed ^18^F-FPTP) for cGMP (current Good Manufacturing Practice) compliance and its biologic validation. The automated module provides consistence, reproducible, and acceptable ^18^F-FTP product.

## 2. Materials and Methods

### 2.1. Synthesis of Cold Fluoropropoxytryptophan (FTP; Reference Standard)

**Figure 1:**
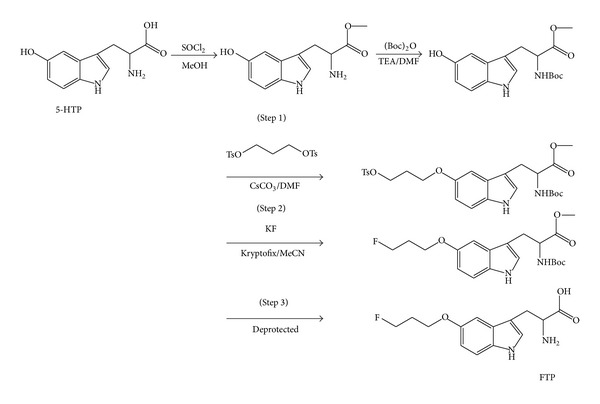
Synthetic scheme of [^18/19^F]fluoropropoxytryptophan (^18/19^F-FTP).

The synthetic scheme of FTP is shown in Figure 1. Three steps were involved in the synthesis. In step 1, thionyl chloride (2.4 mL, 34 mmol) was dissolved in anhydrous methanol (100 mL) and cooled to 0°C. 5-Hydroxytryptophan (5.00 g, 22.7 mmol) was added in portions. After temperature returned to room temperature, the mixture was refluxed overnight. The solvent was evaporated, followed by silica-gel packed column chromatographic purification using ethyl acetate : hexane (4 : 1, v/v) as an eluent to yield 5-hydroxytryptophan methyl ester hydrochloride 5.18 g (84% yield). ^1^H-NMR (300 MHz, CDCl_3_): 7.23 (*d*, *J* = 8.7 Hz, 1 H), 7.15 (*s*, 1 H), 6.91 (*d*, *J* = 2.1 Hz, 1 H), 6.73 (*dxd*, *J* = 8.7, *J*′ = 2.3 Hz, 1 H), 4.29 (*t*, *J* = 6.3, 1 H), 3.83 (*s*, 3 H), and 3.26–3.42 (*m*, 2 H). ^13^C-NMR (300 MHz, CDCl_3_): 169.8, 150.8, 132.2, 127.9, 125.4, 112.2, 112.1, 105.6, 102.1, 53.5, 52.7, and 26.7.

Subsequently, 5-hydroxytryptophan methyl ester (2.4 g, 10 mmol) was dissolved in 100 mL of anhydrous DMF. Anhydrous triethyl amine (4 mL, 30 mmol) was added while stirring. Ditertbutyldicarbonate (2.6 g, 12 mmol) was then added to the solution. The mixture was stirred and heated at 60°C overnight. The reaction mixture was evaporated under reduced pressure and was reconstituted in ethyl acetate. The crude product solution was loaded on a silica gel column and eluted with ethyl acetate : hexane (from 1 : 3 to 2 : 3, v/v). N-Boc-5-Hydroxytryptophan methyl ester was collected after solvent evaporation to yield 3.08 g (92% yield). ^1^H-NMR (300 MHz, CDCl_3_): 7.05 (*d*, *J* = 8.7 Hz, 1 H), 6.90 (*s*, 1 H), 6.84 (*d*, *J* = 2.1 Hz, 1 H), 6.56 (*dxd*, *J* = 8.7, *J*′ = 2.3 Hz, 1 H), 4.30 (*t*, *J* = 6.3, 1 H), 3.56 (*s*, 3 H), 2.90–3.10 (*m*, 2 H), and 1.29 (*s*, 9 H). ^13^C-NMR (300 MHz, CDCl_3_): 173.8, 156.8, 150.4, 132.0, 128.4, 124.2, 111.8, 111.6, 109.0, 102.5, 79.7, 55.0, 51.6, 27.9, and 27.7.

In step 2, an aliphatic tosyl chain was to be added at phenolic hydroxyl group of N-Boc-5-hydroxytryptophan methyl ester. Cesium carbonate (3.9 g, 12 mmol) was added to N-Boc-5-hydroxytryptophan methyl ester (3.4 g, 10 mmol) in 60 mL of anhydrous DMF while stirring under nitrogen atmosphere. 1,3-Ditosyl-propanol (6.2 g, 16 mmol) was added to the solution. The mixture was stirred for overnight at 60°C. The reaction mixture was evaporated under reduced pressure and was reconstituted in ethyl acetate. The crude product solution was loaded on a silica gel column and eluted with ethyl acetate : hexane (from 1 : 3 to 2 : 3, v/v) to yield 4.6 g (84% yield) of N-Boc-tosylpropoxytryptophan methyl ester. ^1^H-NMR (300 MHz, CDCl_3_): 7.77 (*d*, *J* = 8.3 Hz, 2 H), 7.24 (*d*, *J* = 8.8 Hz, 1 H), 7.19 (*d*, *J* = 8.3 Hz, 2 H), 6.97 (*s*, 1 H), 6.91 (*d*, *J* = 2.4 Hz, 1 H), 6.70 (*dxd*, *J* = 8.8, *J*′ = 2.4 Hz, 1 H), 4.53 (*m*, 1 H), 4.28 (*t*, *J* = 6.3 Hz, 2 H), 4.00 (*t*, *J* = 5.8 Hz, 2 H), 3.67 (*s*, 3 H), 3.21 (*d*, *J* = 5.2, 2 H), 2.34 (*s*, 3 H), 2.13 (quintet, *H* = 6.0 Hz, 2 H), and 1.41 (*s*, 9 H). ^13^C-NMR (300 MHz, CDCl_3_): 173.2, 155.7, 153.4, 145.1, 133.3, 131.9, 130.2, 128.4, 128.2, 124.1, 113.1, 112.2, 110.3, 102.2, 77.6, 67.8, 64.4, 54.5, 52.7, 29.5, 28.7, 28.5, and 21.9. Mass spectrometry (M^+^): 545.5 (100%), 445.4 (5%), 171.2 (10%), and 105.2 (10%).

In step 3, N-Boc-tosylpropoxytryptophan methyl ester was reacted with potassium fluoride (KF) in 2,2,2-kryptofix, followed by deprotection. N-Boc-Tosylpropoxytryptophan methyl ester (50 mg, 0.09 mmol), potassium fluoride (KF, 10.5 mg, 0.18 mmol), and 2,2,2-kryptofix (34 mg, 0.09 mmol) were dissolved in 5 mL of anhydrous acetonitrile while stirring under nitrogen atmosphere and was refluxed for 4 hrs. The reaction mixture was evaporated under reduced pressure, reconstituted in ethyl acetate (EtOAc, 0.5 mL), and purified with column chromatography using hexane : EtOAc = 1 : 1(v/v) to obtain 15 mg (43% yield) of N-Boc-fluoropropoxytryptophan methyl ester. ^1^H-NMR (300 MHz, CDCl_3_): 7.16 (*d*, *J* = 8.8 HJz, 1 H), 6.95 (*s*, 1 H), 6.91 (*d*, *J* = 2.3 Hz, 1 H), 6.78 (*dxd*, *J* = 8.8, *J*′ = 2.3 Hz, 1 H), 5.02 (*d*, 2 H), 4.69 (*t*, *J* = 5.9 Hz, 1 H), 4.53 (*t*, *J* = 5.9 Hz, 1 H), 4.07 (*d*, *J* = 5.1 Hz, 2 H), 3.61 (*s*, 3 H), 3.21 (*d*, *J* = 5.1, 2 H), 2.17 (quintet, *H* = 6.0 Hz, 1 H), 2.08 (quintet, *H* = 6.0 Hz, 1 H), and 1.35 (*s*, 9 H). ^13^C-NMR (300 MHz, CDCl_3_): 173.2, 155.7, 153.4, 131.9, 128.5, 124.0, 113.3, 112.3, 110.4, 102.4, 82.5, 80.3, 64.9, 54.7, 52.6, 32.9, 31.2, and 28.7. Mass spectrometry (M^+^): 393.3 (50%), 248.8 (10%), 172.8 (20%), 140.8 (30%), and 112.6 (100%).

To deprotect amino and ester groups, N-Boc-fluoropropoxytryptophan methyl ester (15 mg, 0.038 mmol) was dissolved in dichloromethane : anhydrous trifluoroacetic acid (0.25 mL/0.25 mL). The mixture was stirred for 60 min. After solvent evaporation, 0.5 mL of 1 N NaOH was added to the residue. The reaction mixture was heated at 85°C for 60 min or till full dissolving of the mixture. The product was purified by prep-TLC (1 mm) using MeOH : EtOAC (1 : 3, v : v) to yield FTP (5.5 mg, 44%). ^1^H-NMR (300 MHz, CDCl_3_): 6.97 (*s*, 1 H), 6.91 (*d*, *J* = 2.4 Hz, 1 H), 6.70 (*dxd*, *J* = 8.8, *J*′ = 2.4 Hz, 1 H), 4.53 (*m*, 1 H), 4.28 (*t*, *J* = 6.3 Hz, 2 H), 4.00 (*t*, *J* = 5.8 Hz, 2 H), 3.67 (*s*, 3 H), 3.21 (*d*, *J* = 5.2, 2 H), and 2.13 (quintet, *H* = 6.0 Hz, 2 H). ^13^C-NMR (300 MHz, CDCl_3_): 173.2, 153.4, 131.9, 128.5, 124.0, 113.3, 112.3, 110.4, 102.4, 82.5, 64.9, 54.7, 32.9, and 31.2.

### 2.2. Development of the Automated Module

We have designed and built an automated module which is compact and easy to use and be maintained. This module consists of an apparatus and a control box. This system uses valves as well as other fittings and will be housed in the lead-shielded hot cell. In addition to the main unit, a built-in vacuum pump is used to generate sufficient vacuum for waste collection and venting, and the infrared heater is installed for rapid heating. The control box hosting digital/analog input/output (I/O) modules is operated by LabView software (National Instruments, Austin, TX). We used this software to write the customized user interface for controlling and monitoring the synthesis of ^18^F-FTP. The module provides a rapid synthesis of ^18^F-FTP and can recover ^18^O-enriched water using an anionic resin which is cost-effective.

### 2.3. Radiosynthesis of [^18^F]Fluoropropoxytryptophan (^18^F-FTP)

The synthetic scheme is the same as that of cold FTP (shown in Figure 1). [^18^F]Fluoride was produced by proton irradiation of enriched [^18^O]-water (Sigma Chemical Company, St. Louis, MO) in a small-volume silver target. Aliquots containing 4.44 GBq of ^18^F activity were combined with 26 mg kryptofix-2,2,2 and 4.6 mg anhydrous potassium carbonate, heated under reduced pressure to remove the excess [^18^O]-water, and dried by azeotropic distillation with acetonitrile (3 × 1.5 mL). K^18^F/kryptofix complex was reconstituted in 0.3 mL acetonitrile. An aliquot of K^18^F/kryptofix (0.62 GBq in 0.1 mL acetonitrile) was then administered to the module via an external port. N-Boc-Tosylpropoxytryptophan methyl ester (5 mg) was dissolved in acetonitrile (0.2 mL) and injected through a 1 mL syringe to a reaction vial (RV1). The IR heater automatically warmed the RV1 at 90°C for 15 min. The mixture in the RV1 was passed through a silica gel packed column (SPE-500 mg, Whatman Lab, Clifton, NJ) and eluted with 1 mL ethyl acetate to the reaction vessel 2 (RV2) under nitrogen flow to remove free fluoride. Before de-BOC, the solution inside the RV2 was evaporated under vacuum at 90°C for 15 min. After that, trifluoroacetate (0.2 mL) in dichloromethane (0.4 mL) was loaded into the RV2 to deprotect amino group. The solution was set under room temperature for 10 minutes to allow the reaction to complete and then the solvent was evaporated to dryness under vacuum for 15 minutes. For deesterification, ethyl alcohol (0.4 mL) and 1 N NaOH (0.2 mL) were added into RV2. The hydrolysis of the ester group was performed at 90°C for 15 min. The solution in RV2 was evaporated under reduced pressure. ^18^F-FTP was reconstituted in water (1.2 mL) and filtered through a 0.22 *μ*m filter. The activities of column, RV1 and RV2, were counted upon completion of the synthesis. A NIST-traceable dose calibrator was used to determine total activity (GBq or mCi) and the radioactivity concentration (GBq/mL or mCi/mL). A radio thin-layer chromatographic (radio-TLC) scanner (Bioscan System 200, Washington, DC) and high performance liquid chromatography (HPLC) using a 20 *μ*L loop and Bondapak CN-RP column eluted with methanol : water = 7 : 3, v/v and flow rate 0.5 mL/min at 210 nm were performed to assure the purity and identity of ^18^F-FTP. To assure the quality of the product, the pyrogenicity test using Bacterial Endotoxins kit (Sigma, St. Louis, MO), the pH (by pH paper), and residual solvents (by LC-MS) were measured. The sterility test using aerobic (NR6) and anaerobic (NR7) fermentation broth (Becton Dickinson Diagnostic Instrument Systems, Towson, MD) were conducted at 37°C for 7 days. The cloudiness of the broth was examined. The membrane filter integrity was also examined.

### 2.4. *In Vitro* Cellular Kinetic Assay

To assure quality of ^18^F-FTP, cell uptake assays were performed for biologic validation. Cell uptake assay of ^18^F-FTP and ^18^F-FDG was performed using human prostate cancer cells (PC-3) as described in known literature. The cell line was selected because it is known as an excellent* in vitro* model for studying the interaction of large neutral amino acid conjugated drugs with LAT1 transporter [22]. [^3^H]-Tyrosine kinetic assays showed the saturable kinetics with K(m) and V(max) values of 34 ± 3 *μ*M and 0.70 ± 0.02 nanomoles/min/mg protein, respectively. The cell line was purchased from American Type Tissue Culture (Bethesda, MD). Cells were maintained in the mixtures of Dulbecco's modification of Eagle's medium (DMEM), F-12 (GIBCO, Grand Island, NY), and 10% phosphate buffered saline (PBS) at 37°C in a humidified atmosphere containing 5% CO_2_. The cells were plated to 12-well tissue culture plates that contained 50,000 cells per well. The cells in each well were incubated with 0.74 MBq of ^18^F-FTP or ^18^F-FDG at 37°C for 30–120 min, respectively. After incubation, cells were washed with ice-cold phosphate-buffered saline (PBS) twice and trypsinized with 0.5 mL of trypsin solution. Then, cells were collected and the radioactivity was measured by gamma counter (PerkinElmer, Waltham, MA). Data are expressed in mean ± S.D. percent uptake of three measurements.

### 2.5. PET Imaging Studies

The animals were housed in the University of Texas M. D. Anderson Cancer Center facility. All protocols involving animals were approved by the M. D. Anderson Animal Use and Care Committee. Expression of LAT1 in neuroendocrine tumors of the lung has been reported [23]. NCI H187Lu is an endocrine (small cell lung cancer) cancer cells and orthotopic nude mouse model is well established [24]. Thus, it was selected for comparison with nonendocrine (PC-3) mouse model. Athymic nude mice (15–20 g; NCI–NIH, Bethesda, MD) were inoculated with PC-3 or human small cell lung cancer NCI-H187Lu cells (s.c. 10^6^ cells/mouse) at the left legs. After 16 days, a tumor size of 1 cm was observed. Mice were anesthetized with 2% isoflurane and injected intravenously with 7.4 MBq of ^18^F-FDG and ^18^F-FTP, respectively. Four serial 15-minute transaxial PET images were obtained through microPET/CT (Inveon, Siemens Medical System, Malvern, PA) or Concorde R4 microPET scanner (Knoxville, TN). All corrections for attenuation, scatter, dead time, and randoms were applied to generate quantifiable images.

## 3. Results

### 3.1. Chemistry

**Figure 2:**
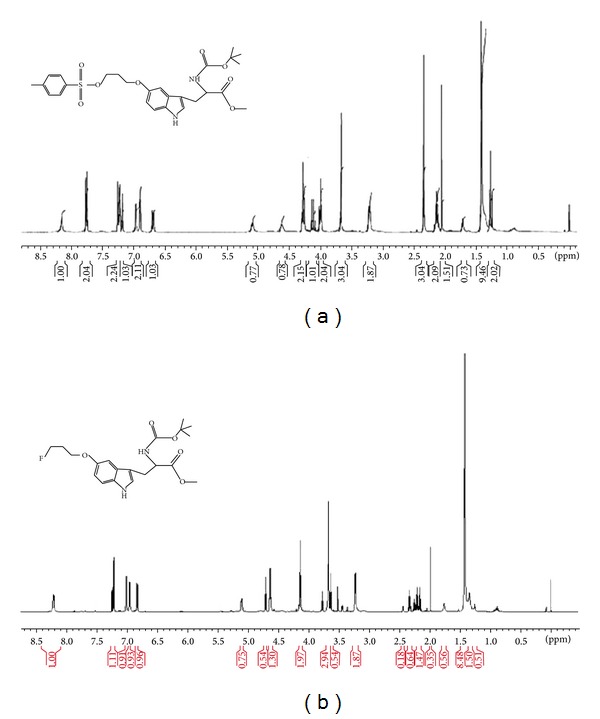
^1^H-NMR of N-Boc-tosylpropoxytryptophan methyl ester (a) and N-Boc-fluoropropoxytryptophan methyl ester (b).

Using N-Boc-tosylpropoxytryptophan methyl ester as a precursor, we are able to shorten the synthetic step to one-step synthesis. ^1^H-NMR of N-Boc-tosylpropoxytryptophan methyl ester and N-Boc-fluoropropoxytryptophan methyl ester are shown in Figure 2.

**Figure 3:**
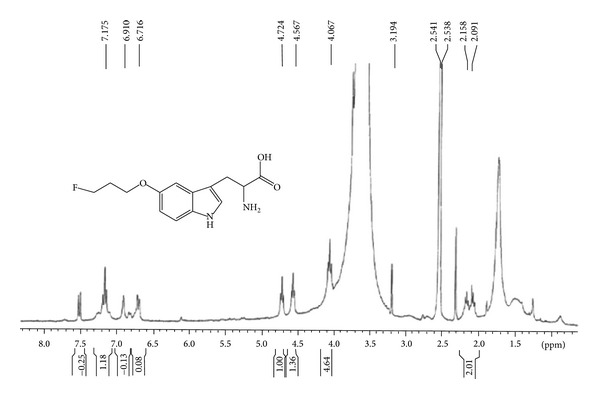
^1^H-NMR of cold fluoropropoxytryptophan (FTP).

**Figure 4:**
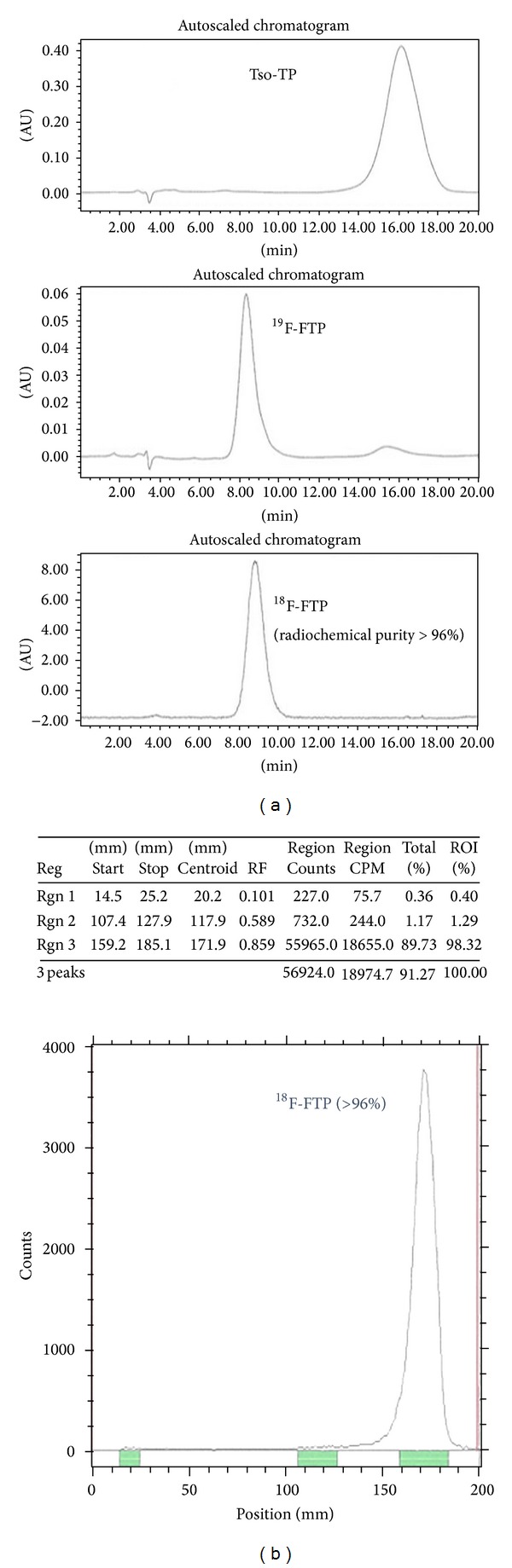
(a) HPLC of ^18^F-FTP showed radiochemical purity >96% using C-18 RP (MeOH : water = 7 : 3) at flow rate 0.5 mL/min; (b) radio-TLC of ^18^F-FTP showed greater than 96% purity using mobile phase ammonium acetate (1 M) : methanol (4 : 1, v : v).

**Figure 5:**
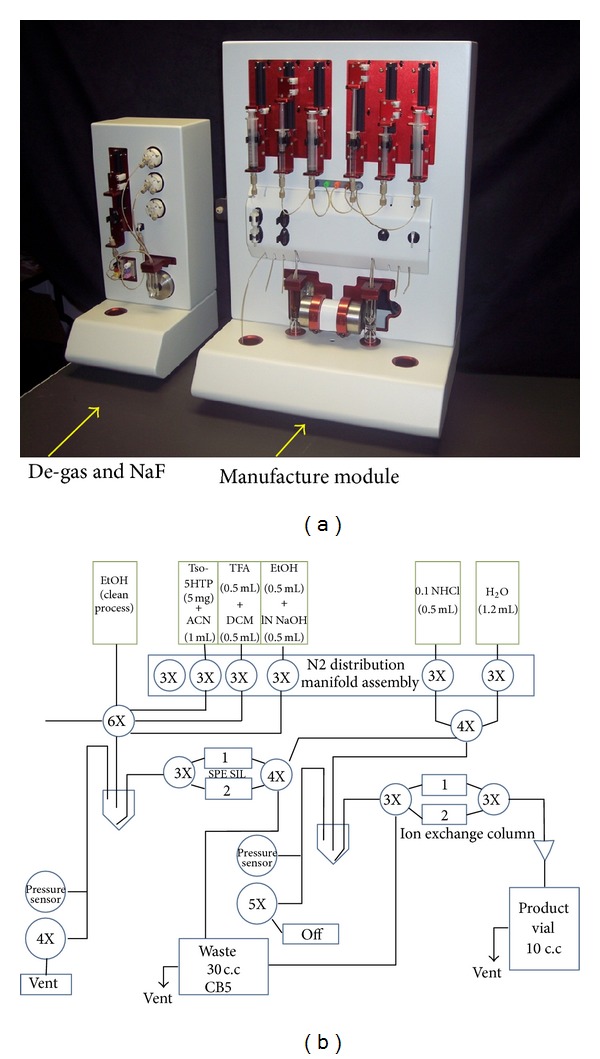
(a) Automated module for production of ^18^F-FTP. (b) Functional component illustration in this module.

After hydrolysis, ^1^H NMR of fluoropropoxytryptophan (FTP) is shown in Figure 3. During radiosynthesis, the activity of N-Boc-^18^F-fluoropropoxytryptophan methyl ester was 0.30 GBq (55.63%, decay-corrected). The radioactivity, radiochemical yield, and end-of-synthesis (EOS) time for ^18^F-FTP were 0.13 GBq, 37.73% (decay-corrected), and 90 min, respectively. Radio-TLC (1 M ammonium acetate : methanol = 4 : 1, v/v) showed that the retarded factor (*R*_*f*_) value was 0.9. HPLC of tosyl precursor and ^18^F-FTP showed that the retention time (*R*_*t*_) was 16 min and 9 min, respectively (Figure 4(a)). The no-carrier-added displacement product corresponded to the unlabeled FTP under the same TLC and HPLC system. Radiochemical purity determined by HPLC and radio-TLC of the title compound was >96% (Figure 4). The specific activity of ^18^F-FTP determined by HPLC was 74 GBq/*μ*mol. The pyrogenicity test (Bacterial Endotoxins) was <175 EU/4 mL and the pH was about 6.5. The product was sterile. There was no corruption for membrane filter integrity. LC-MS showed no more than 0.04% acetonitrile or methylene chloride of residual solvents in ^18^F-FTP product. Automated module for production of FTP and the flow chart of the module are shown in Figure 5.

### 3.2. *In Vitro* Cellular Kinetic Assay

**Figure 6:**
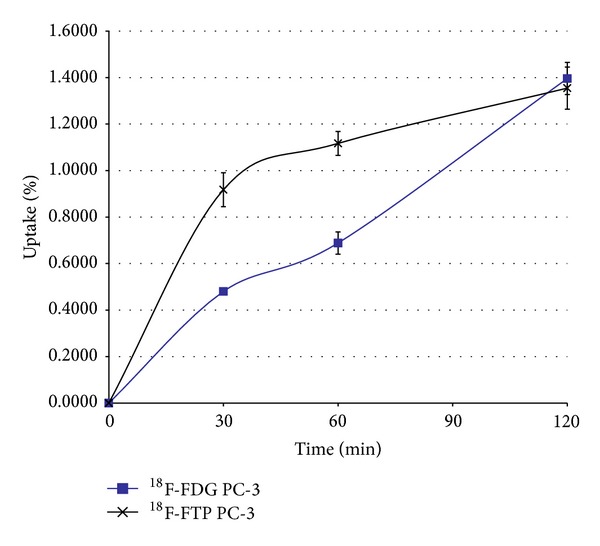
*In vitro* cellular kinetic assays of ^18^F-FTP and ^18^F-FDG showed similar enhanced uptake patterns as a function of incubation time in human prostate cancer cell line PC-3.

*In vitro* cell culture assays of ^18^F-FTP and ^18^F-FDG showed similar enhanced uptake patterns as a function of incubation time in PC-3 prostate cancer cells, and the uptake reached about 1.4% uptake for both radiotracers (Figure 6).

### 3.3. PET Imaging Studies

**Figure 7:**
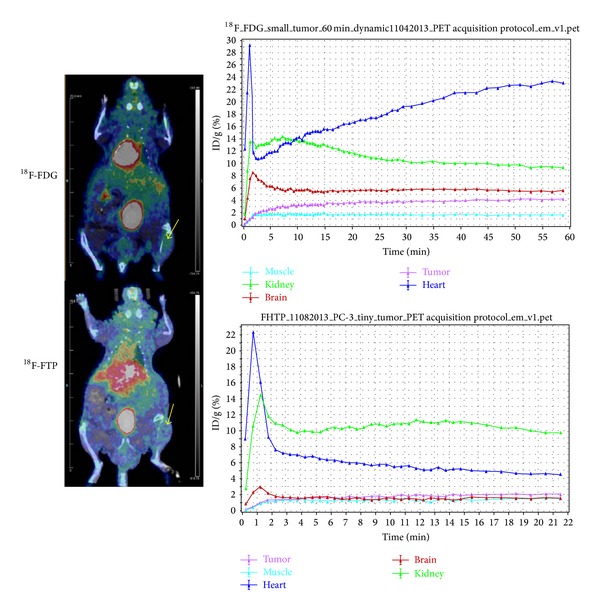
MicroPET/CT showed that ^18^F-FTP had less tumor uptake than ^18^F-FDG in prostate cancer model (PC-3, at 22 min). Arrow: tumor.

**Figure 8:**
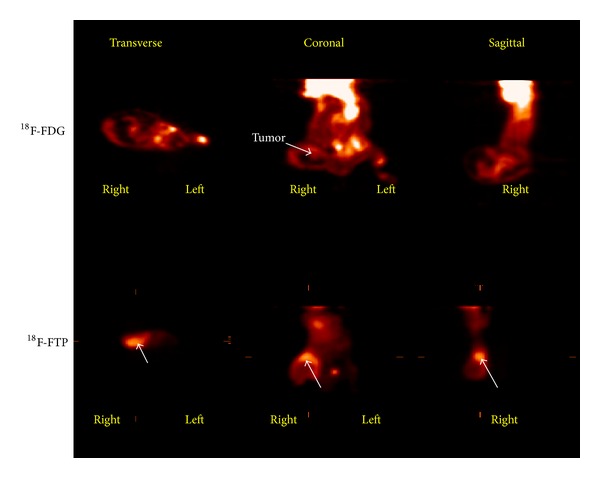
MicroPET imaging in human small cell lung tumor-bearing mice (NCI-H187Lu) at 45 min showed that ^18^F-FTP had more tumor uptake than ^18^F-FDG. Arrow: tumor.

MicroPET/CT imaging studies showed that ^18^F-FTP had less tumor uptake than ^18^F-FDG at 22 min after administration of the tracer in prostate cancer model (Figure 7). However, ^18^F-FTP had more uptake than ^18^F-FDG at 45 min after administration of the tracer in small cell lung cancer model NCI-H187Lu by visualization (Figure 8). The uptake difference could be due to the nature of LAT1 activity in animal models.

## 4. Discussion

Assessment of the effectiveness of cancer therapy (e.g., volumetric and morphological changes) is generally measured by CT and MRI. In addition to these imaging modalities, the treatment endpoints rely almost exclusively on the analysis of biopsies by molecular and histopathological methods. These methods provide a microscopic picture of the general heterogeneous process. On the other hand, nuclear imaging measures blood flow and activity patterns. Therefore, to assess clinical endpoints adequately, a predictive nuclear biomarker is needed that would allow precise measurement of tumor targets on a whole-body image upon administration of a functional radiolabeled agent. These mechanism-based agents provide image-guided therapy which may discontinue ineffective treatment in the earlier phase and may be beneficial to patients.

Transporters for essential amino acids are particularly important because they are involved in protein synthesis to maintain cell integrity and cell cycle progression. Among several amino acid transporters, system L, a Na^+^-independent amino acid transport system, is a major route for providing cells with large neutral amino acids, including branched or aromatic amino acids. Large L-type amino acid transporter 1 (LAT1), a system L subtype, is suggested to be a target for therapy with antiproliferative inhibitors in cancer [25]. LAT family is known to form heterodimers, which contain a chaperone-like heavy chain 4F2hc, and a 12-time transmembrane light chain that is unique to each subtype [26]. LAT1/4F2hc complex, one form of 4F2 antigen or CD98 antigen, preferentially transports large neutral amino acids, such as leucine, isoleucine, valine, phenylalanine, tyrosine, tryptophan, methionine, and histidine, which are essential for cell survival and proliferation [27]. An increasing amount of research shows that LAT1/4F2hc is overexpressed in a variety of human tumor cell lines and tumor tissues, suggesting that LAT1/4F2hc is implicated in the growth and proliferation of multiple types of human cancers [26]. Assessment of LAT1 activity provides potential applications in differential diagnosis and prediction of early treatment response in oncology and autoimmune diseases. Though ^18^F-FDG-PET imaging demonstrates the increased glucose consumption of malignant cells, problems with specificity for cell proliferation have led to the development of new PET tracers. ^18^F-FDG could assess metabolic activity, but it could not adequately assess neuroendocrine activity. ^18^F-Fluorodopa has been used in clinic to assess dopamine transformation in both oncology and neurologic diseases. Due to the nature of LAT1 and neuroendocrine involvement in cancers and neurologic diseases, it would be important to develop a biomarker to probe LAT1 because LAT1 is the rate limiting step for the tumor and neuron uptake. The structure of ^18^F-FTP is the same as ^18^F-FPTP and ^18^F-FPTP uptake is inhibited by LAT1 inhibitor, such as BCH (2-aminobicyclo-(2,2,1)-heptane-2-carboxylic acid)^21^. Thus, cell uptake of ^18^F-FTP is recognized by LAT1.

The key difference in the synthesis of ^18^F-FTP between our work and He et al. was the precursor [21]. The reported manual synthesis by He et al. used a 2-pot synthetic method which caused lower yield [21]. The purity of our precursor (tosylpropoxytryptophan) and reference standard (FTP) were greater than 95%. Using our automated module, the radiochemical purity of ^18^F-FTP was greater than 95%; 99.5% of the observed ^18^F-FTP *γ*-emissions measured by a multichannel analyzer (MCA) corresponded to the 0.511 MeV. The ^18^F-FTP product meets general acceptance criteria such as purity, identity, pH, pyrogenicity, and sterility. The overexpression of amino acid transporter system, such as LAT1, is an important marker for disease prognosis [22, 28]. In microPET imaging study, ^18^F-FTP showed different biodistribution in two different tumor models. ^18^F-FTP had less tumor uptake than ^18^F-FDG at 22 min after administration in a PC-3 prostate cancer mouse model, while exhibiting higher tumor uptake in mice bearing NCI-H187Lu small cell lung cancer at 45 min after injection. The amount of tumor uptake may depend on the status of LAT1 transporter density in the two cancer cell lines. Though the study is focused on the development of an automated synthesis method of ^18^F-FTP which fulfills the cGMP grade, further studies warrant determining the information of the LAT1 transporter density of NCI-H187Lu cancer cells and PC-3 prostate cancer cells.

In summary, ^18^F-FTP was synthesized efficiently by one-pot method with high radiochemical yield and purity using an automated module.* In vitro* cellular uptake and PET imaging studies confirm biologic validation. Thus, ^18^F-FTP may improve the diagnosis, planning, and monitoring of tryptophan transporter pathway-directed therapies.
